# Effect of chewing an apple on dental plaque removal and on salivary bacterial viability

**DOI:** 10.1371/journal.pone.0199812

**Published:** 2018-07-18

**Authors:** Susana Rubido, Lucía García-Caballero, María Teresa Abeleira, Jacobo Limeres, Marta García, Pedro Diz

**Affiliations:** Medical-Surgical Dentistry Research Group (OMEQUI), Health Research Institute of Santiago de Compostela (IDIS), University of Santiago de Compostela (USC), Santiago de Compostela, Spain; University of Brescia, ITALY

## Abstract

**Objectives:**

Studies on dental plaque removal by chewing an apple are scarce and dated, with conflicting findings. This study aimed to determine whether chewing an apple produced mechanical removal of dental plaque or had any effect on salivary bacterial viability.

**Methods:**

The study group consisted of 20 healthy adults with good oral health status who were randomly assigned to brush their teeth or eat an apple. After 2 weeks, the experiment was repeated with the order reversed. Plaque index (PI) and the bacterial viability (BV) in a sample of whole saliva (spit) were determined before brushing or apple eating (baseline, B), immediately afterward (A) and 24 hours afterward (24).

**Results:**

After chewing an apple, PI-A was significantly higher than both PI-B (P < .001) and PI-24 (P < .001). BV-A was significantly lower than BV-B (P < .001), with a return to baseline values at the BV-24 measurement.

**Conclusions:**

Chewing an apple does not remove dental plaque, and may favor plaque regrowth during the first 24 hours, but it does produce an immediate reduction in salivary bacterial viability similar to that after tooth brushing.

## Introduction

It has traditionally been advocated that ending a meal with a hard food or fruit is a means to prevent oral diseases such as caries and periodontal disease [1, 2]. Apples have been commonly recommended as a means of cleaning the teeth after eating because they stimulate an alkaline saliva flow that neutralizes the acids produced in dental plaque after carbohydrate ingestion [1]. As a result, apples were included in health-education programs [3], to the extent that they became synonymous with oral and dental health [4, 5], leading to popularisation of the saying that “An apple a day keeps the dentist away”.

Studies performed during the 1960s and 70s suggested that chewing an apple did not have any effect on the elimination of plaque [6,7]. However, discrepant findings were reported in a paper published in 1986 by Schneider and Knieknecht [8], who stated that chewing an apple significantly reduced the accumulation of plaque, although its efficacy was half that achieved with supervised tooth brushing.

This controversy was explained by the assertion that chewing fibrous foods provided a degree of dental cleaning except in areas of difficult access, such as the interdental spaces and regions close to the gingival border [2, 9]. However, to further complicate the issue, it must not be forgotten that, apart from their high sugar content, apples are highly acidic and provoke a marked fall in the pH of plaque, with the risk that this carries for the tooth surfaces [10]. Eating apples on a daily basis has thus been associated with tooth wear in dentine, probably because this acidity could lead to early dentine exposure [11].

We have found no studies published in recent decades that have shown definitively whether or not eating apples has an anti-plaque effect. We therefore designed this study to evaluate whether chewing an apple produced mechanical removal of plaque or affected plaque regrowth, and whether it had any effect on the vitality of bacteria that colonise the saliva.

## Material and methods

### Selection of the study group

The sample size calculation was performed “a priori” using the G*Power 3.1.5. software [12]. The following criteria were established: effect size of 0.35, alpha error of 0.05, and statistical power of 80%. Assuming these criteria, a sample size of at least 15 subjects was required. To ensure a sufficient number of participants completed the study, we selected 20 healthy adult volunteers aged 20–25 years, who were students at the School of Dentistry of the University of Santiago de Compostela, Spain. All participants presented a good baseline oral health status based on the following parameters: minimum of 24 examinable permanent teeth (excluding third molars and teeth with fixed prostheses or extensive restoration), with no evidence of gingivitis or periodontitis (Community Periodontal Index score = 0) [13], and an absence of active caries. The following exclusion criteria were applied: smoking, presence of removable dental prostheses or orthodontic appliances, antibiotic treatment or the routine use of oral antiseptics during the previous three months, and presence of any systemic disease that could affect the production and/or composition of the saliva.

The study design complied with the Helsinki Declaration on Ethical Principles for Medical Research Involving Human Subjects and was independently reviewed and approved by the Ethics Committee of the University of Santiago de Compostela, Spain (register number 917/2013). All participants were informed about the study and gave their written consent to participate in it.

### Study design

Three days before starting the study, the teeth of all participants were given a professional scaling and prophylaxis (S & P). The measurements of plaque and bacterial vitality were always performed in the morning (between 9 am and 12 noon). Participants were not allowed to use any oral hygiene technique for 24 hours prior to starting each experiment, or to consume alcohol or foods that could promote mechanical removal of plaque, although they were asked otherwise to maintain their normal dietary habits. Each volunteer underwent two different experiments in which they performed distinct plaque removal techniques: manual tooth brushing (MT) or chewing an apple (APP). The order of the experiments was determined using a balanced randomisation system, and there was a two-week rest period between each experiment.

MT was performed using only a Vitis^®^ medium toothbrush (Dentaid, Barcelona, Spain) and sterile water (10 mL), with no toothpaste. The Bass tooth brushing technique was employed for a total of two minutes (30 seconds per quadrant of the oral cavity). All the apples used were of the Golden Delicious variety, with a mean weight of 160g; mastication was performed alternating between the two sides of the mouth, but no time limit was imposed.

The plaque index (PI) was determined at three time points during each experiment: at baseline (PI-B), on arrival of the participant at the clinic; immediately after performing the plaque removal technique (PI-A); and 24 hours after completing the removal technique (PI-24). Non-stimulated saliva samples were taken at these same three time points to determine bacterial vitality (BV) in the saliva: at baseline (BV-B), immediately after performing the removal technique (BV-A), and at 24 hours (BV-24).

For 24 hours after completing the removal technique (until after the PI-24 and BV-24 measurements), participants did not perform their routine oral hygiene techniques, drink alcohol or eat food that could promote mechanical plaque removal, although they did otherwise maintain their usual dietary habits.

### Macroscopic quantification of dental plaque

The presence of plaque on the tooth surfaces was determined by visual inspection of erythrosine-stained plaque (Plac Control^®^, Dentaid, Barcelona, Spain) at six sites per tooth [14]: mesiovestibular, mediovestibular, distovestibular, mesiolingual, mediolingual and distolingual. The Turesky modification of the Quigley-Hein plaque index [15, 16] was used to quantify plaque. The assessment was performed by a single examiner. The Quigley-Hein PI [15] modified by Turesky [16] is a previously validated index and is one of the most widely used indices to evaluate oral hygiene products in which plaque is quantified [17, 18, 19].

The mean PI for each subject was determined as the sum of all the individual indices (6 per tooth) divided by the total number of measurements (number of scorable teeth multiplied by 6). The PI of the vestibular surfaces was defined as the sum of the individual indices (3 per tooth) of the superior and inferior vestibular surfaces divided by the total number of measurements (number of scorable teeth multiplied by 3). The PI of the palatal/lingual surface was defined as the sum of all the individual indices (3 per tooth) of the palatal and lingual surfaces divided by the total number of measurements (number of scorable teeth multiplied by 3).

At the end of each experiment, all tooth surfaces were cleaned professionally to remove remaining supragingival plaque and the plaque-staining solution.

### Analysis of bacterial vitality in saliva samples

The antibacterial activity of tooth brushing and of chewing an apple was analysed by evaluation of the vitality of salivary bacteria using an epifluorescence microscopy technique (LIVE/DEAD^®^BacLight^™^, Molecular Probes, Leiden, The Netherlands). The fluorescence solution was formed of two fluorochromes, SYTO-9 and propidium iodide, with excitation/emission maxima of 480/500 nm for SYTO-9 and of 490/635 nm for propidium iodide. The simultaneous application of the two fluorochromes enables bacteria with intact cytoplasmic membranes (green fluorescence) to be differentiated from bacteria with damaged membranes (red fluorescence). The LIVE/DEAD^®^BacLight^™^ fluorescence solution was prepared in accordance with the manufacturer’s instructions in 5 mL of sterile water filtered through a 0.22 μm Millipore membrane (Millipore Iberica S.A., Madrid, Spain), with a one-to-one ratio of the two fluorochromes. The solution was stored at -20°C.

Non-stimulated saliva samples were collected using a previously described spitting method [20]: participants were instructed to swallow before beginning the collection, and were then told to collect saliva behind closed lips and expectorate into a test tube at the end of each minute of a 3-minute trial. Then, saliva samples were centrifuged at 2000 rpm for 6 minutes. The pellet was resuspended in 100 μL of sterile water and shaken to obtain a homogeneous suspension before adding the fluorescence solution (100 μL). The suspension was stored in the dark at room temperature for 15 minutes and examined using an Olympus BX 51 microscope (Olympus, Tokyo, Japan) fitted with a filter set for fluorescein and Texas Red. The count of viable and non-viable bacteria was performed at high power (x100) on 20 microscope fields (10 fields per slide) that presented a minimum of 100 bacteria (excluding bacterial aggregates). The mean percentage of bacterial viability was calculated for each saliva sample.

### Statistical analysis

The results were analysed using the SPSS version 15.0 statistical package for Windows (SPSS Inc, Chicago, USA). All the PI and BV values showed a normal distribution (Kolmogorov-Smirnoff test). Joint analysis of the influence both of the time point of the measurements and of the experiment undertaken was performed using mixed models with two fixed effects (time and experiment) and one random effect (each participant). A repeated-measures ANOVA test for data with a gamma distribution was used to determine whether differences existed in the PI detected on the different tooth surfaces (for example, vestibular versus lingual/palatal).

## Results

### Assessment of dental plaque

**Fig 1:**
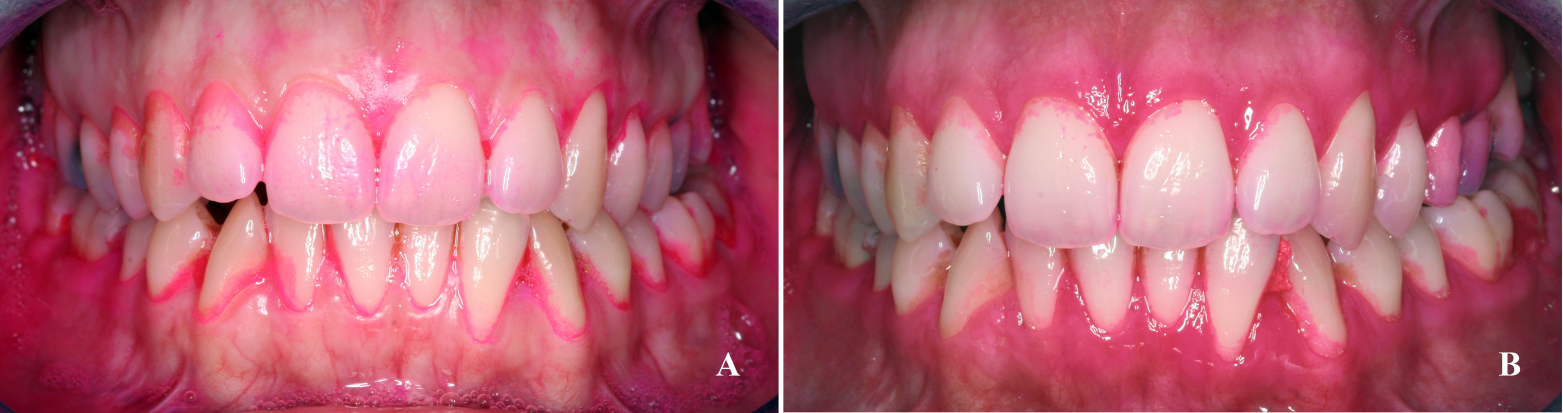
Effect of tooth brushing with sterile water on the dental plaque index. (A) Plaque stain at baseline. (B) Plaque stain immediately after tooth brushing with sterile water.

**Fig 2:**
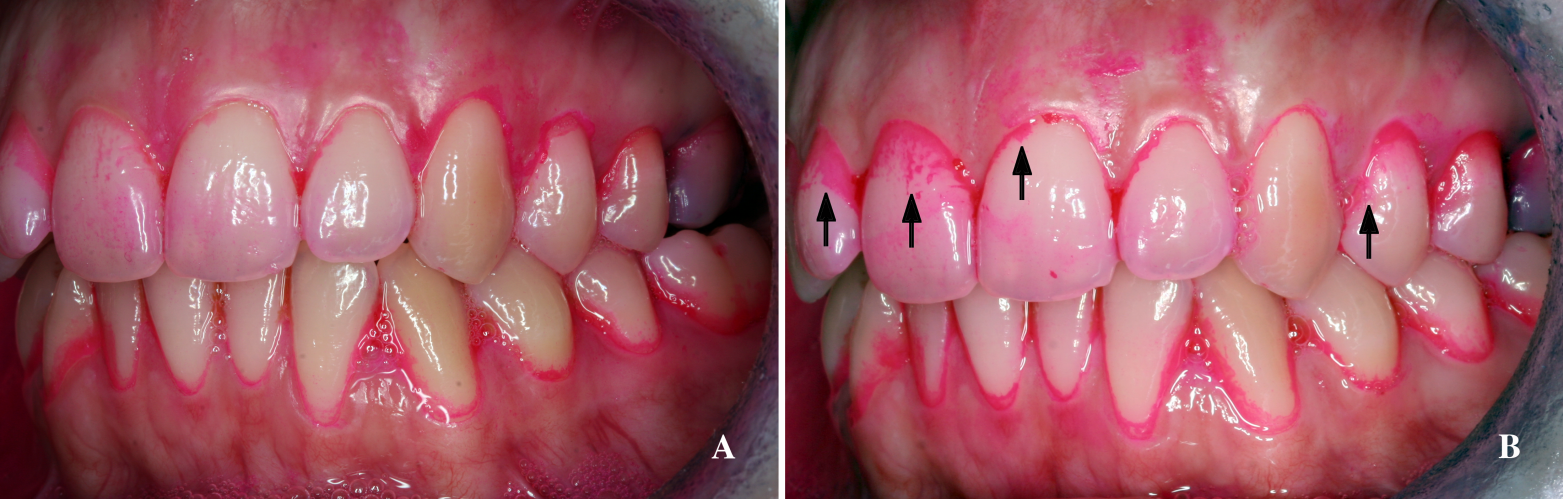
Effect of chewing an apple on the dental plaque index. (A) Plaque stain at baseline. (B) Plaque stain immediately after chewing an apple.

**Fig 3:**
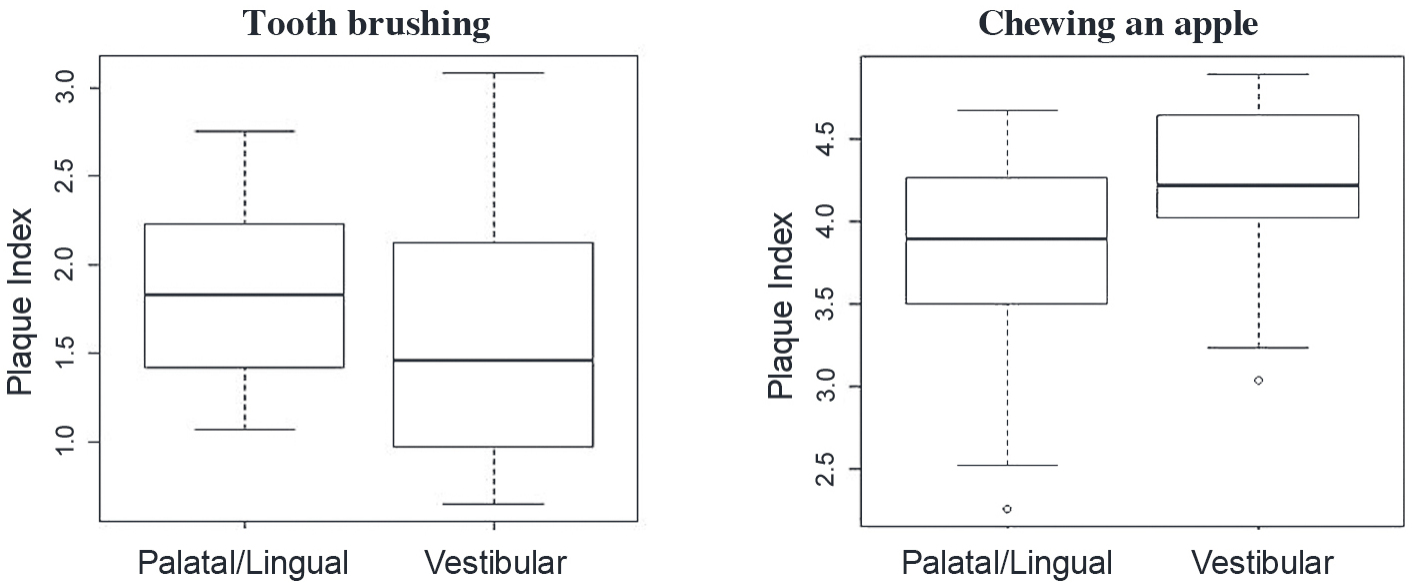
A box plot showing plaque index reduction on vestibular and on palatal/lingual surfaces after tooth brushing and apple eating. Tooth brushing reduced the plaque index more on vestibular than on palatal/lingual surfaces (*P* = 0.002), whereas apple eating reduced the plaque index less on vestibular surfaces than on palatal/lingual surfaces (*P*<0.001).

The mean PI value after tooth brushing was lower than before tooth brushing (Fig 1 and Table 1), whereas after chewing an apple the mean PI was higher than before tooth brushing (Fig 2 and Table 1). On comparing the two methods of plaque removal, it was found that tooth brushing produced significantly lower PI values immediately and 24 hours after cleaning than chewing an apple (Table 1). Tooth brushing reduced PI more on vestibular than on palatal/lingual surfaces, whereas apple eating reduced PI less on vestibular surfaces than on palatal/lingual surfaces (Fig 3).

**Table 1:** Plaque indices at baseline, immediately after manual tooth brushing and after chewing an apple, and 24 hours after the activity (N = 20).

	PI-B mean (range)	PI-A mean (range)	PI-24 mean (range)	Statistical significance PI-A vs PI-B *P*-value	Statistical significance PI-24 vs PI-A *P*-value	Statistical significance PI-A MT vs APP *P*-value	Statistical significance PI-24 MT vs APP *P*-value
**MT**	2.338 (1.100–3.130)	1.743 (0.930–2.910)	2.575 (1.800–3.700)	<0.001	<0.001	<0.001	<0.001
**APP**	2.849 (1.900–3.850)	3.998 (2.740–4.780)	3.333 (2.560–4.140)	<0.001	<0.001		

Abbreviations: MT, manual tooth brushing; APP, chewing an apple; PI-B, baseline plaque index; PI-A, plaque index immediately after performing the activity; PI-24, plaque index 24 hours after performing the activity

### Bacterial vitality in saliva

The mean BV-A value after tooth brushing was lower than before tooth brushing (Table 2). After chewing an apple, the mean BV-A was also lower than before chewing an apple (Table 2). The mean values of BV-24 after tooth brushing and after chewing an apple were significantly higher than the BV-A values in both cases (Table 2). On comparing the two methods of plaque removal, it was found that BV-24 was significantly higher after chewing an apple than after tooth brushing (Table 2).

**Table 2:** Bacterial vitality at baseline, immediately after manual tooth brushing and after chewing an apple, and 24 hours after the activity (N = 20).

	BV-B % mean (range)	BV-A % mean (range)	BV-24% mean (range)	Statistical significance BV-A vs BV-B *P*-value	Statistical significance BV-24 vs BV-A *P*-value	Statistical significance BV-24 MT vs APP *P*-value
**MT**	91.25 (86.00–97.00)	62.21 (55.00–70.70)	80.26 (69.00–89.40)	<0.001	<0.001	0.009
**APP**	82.15 (72.80–89.90)	60.77 (49.60–81.20)	87.11 (76.40–95.00)	<0.001	<0.001	

Abbreviations: MT, manual tooth brushing; APP, chewing an apple; BV-B, baseline bacterial vitality; BV-A, bacterial vitality immediately after performing the activity; BV-24, bacterial vitality 24 hours after performing the activity.

## Discussion

Tooth brushing is effective in reducing levels of dental plaque [21], thus tooth brushing was the reference technique for mechanical control of plaque. After performing the tooth brushing, a significant reduction in the plaque index was observed with respect to the baseline plaque index. Plaque elimination was greater on vestibular than on palatal/lingual surfaces, as has been demonstrated previously by other authors [8]. Bacterial vitality in saliva immediately after performing the tooth brushing_using only sterile water with no toothpaste_was significantly reduced with respect to the baseline determination, confirming the results of previous studies [19].

The apple is popularly considered to be the gold standard of foods that are able to remove food residues and plaque [3, 4]. Although this belief is still widely held in many circles, some authors have indicated that apples have little or no capacity to remove plaque [6, 8, 9]. Discrepancies between the various studies that have evaluated the efficacy of mechanical plaque removal by chewing an apple could be due to methodological differences such as the age of the participants [6, 8], the teeth evaluated [7, 8], the variety of apple used [6, 22], the quantity of apple used [6, 8] or the use of whole or peeled apples [6, 7].

In the present study, chewing an apple not only did not reduce the PI, but the score actually increased. In an attempt to explain this paradoxical finding, a volunteer underwent an additional experiment. He first performed tooth brushing using a conventional technique, with no time limit, until complete removal of all the remaining plaque (no macroscopic evidence of stained material on the tooth surfaces after the administration of a plaque-disclosing tablet). He then chewed a Golden Delicious apple (with no time limit), alternating mastication between the two sides of the mouth. After finishing the apple, another plaque-disclosing tablet was administered and the surface of the teeth was observed to be covered by a discrete biofilm. This experiment demonstrated that apples must contain some component capable of adhering to the tooth surface that stains with erythrosine; another plausible explanation is that chewing the apple stimulates the flow of saliva, and this background staining is caused by erythrosine staining the salivary protein pellicle on the enamel surface.

In our study, the PI-24 was higher than the PI-B, with greater accumulation on the vestibular surfaces than on the palatal/lingual surfaces, particularly on the upper teeth, which have greater contact with the apple. To date we have only found one study in which short-term plaque regrowth has been studied. Working in a school setting over a 10-week period, Longhurst and Berman [22] found that finishing the midday meal with an apple provoked an increase in plaque accumulation.

In this study we found a significant fall in bacterial viability immediately after chewing an apple. This result could have been induced by increased salivary flow and by alkalinisation of the saliva, which would increase the lavage effect and reduce the bacterial concentration per millilitre of saliva. We have been unable to find any published studies that have used bacterial viability to evaluate the antibacterial activity of apple on salivary flora. However, in the past two decades numerous studies have been published on the anti-cariogenic effect of polyphenols extracted from different types of plants [23, 24]. *In vivo* studies performed in humans have shown that the polyphenols present in apples effectively inhibit plaque formation [25]. It has been suggested that these polyphenols may have significant anti-cariogenic properties as they inhibit glucosyltransferase activity and bacterial adherence [26]. The antibacterial effects of the polyphenols [27] or other components of apple should therefore also be taken into account.

In our study, bacterial vitality in saliva at 24 hours after eating an apple had returned to baseline levels; this did not occur with tooth brushing. After finishing eating an apple, the alkaline pH of the saliva is not maintained, and the acidity of the apple itself and of food residues present in the oral cavity create an ideal environment for the proliferation not only of salivary bacteria but also of bacteria that make up the dental plaque biofilm. In addition to the residual plaque left by ineffective mechanical removal by the apple, the higher salivary bacterial concentration will contribute to plaque regrowth [28].

This study has certain limitations that must be taken into account. It may not be appropriate to extrapolate the results to other age groups or to other populations of similar age but of different socio-cultural origin. The most widely used model in published studies of plaque regrowth is the so-called “4-day plaque regrowth model” [29], but the 24-hour model employed in the present study is more likely to reflect the real-life setting and has been shown to be suitable for the evaluation of potential plaque-inhibiting agents [19].

In conclusion, chewing an apple does not necessarily have a mechanical plaque removal effect. In fact, there is an immediate increase in the tooth surface stained by the plaque-disclosing agent, although this is probably because the fruit contains some component able to adhere to the tooth surface and that stains with erythrosine. Apple produces an immediate reduction in salivary bacterial vitality, similar to that achieved with tooth brushing. In terms of plaque index and bacterial vitality, apple is associated with greater plaque regrowth than tooth brushing over a period of at least 24 hours.

## Supporting information

S1 Consort Consort 2010 checklist of the present study. (DOC)

S1 Table Report of the primary data. (SAV)
